# HIV-2 goes global: an unaddressed issue in Indian anti-retroviral programmes

**Published:** 2010-12

**Authors:** Thushan de Silva, Robin A. Weiss

**Affiliations:** * MRC/UCL Centre for Medical Molecular Virology, Division of Infection & Immunity, University College London; United Kingdom; ** Medical Research Council (UK) Laboratories Atlantic Road, PO Box 273, Fajara, The Gambia

In this issue, a report by Chiara *et at*^1^ is one of the first to originate from India on the outcomes of antiretroviral therapy (ART) in HIV-2 and HIV-1/2 dually infected patients. They describe the proportions, baseline characteristics and outcomes of ART in HIV-1, HIV-2 and HIV-1/2-infected individuals managed in an urban referral clinic in Mumbai. Since the first case of HIV-2 from India was reported in 1991, others have been identified from geographically diverse states, yet reliable and up-to-date information on the HIV-2 epidemic in India is still lacking^2^,^3^. Sequential serological surveys from a hospital population in Tamil Nadu performed during 1993 - 1997 and 2000 - 2001 showed a stable HIV-2 prevalence over time, at 2.47 per cent of all HIV diagnoses at the latter time point, equating to 0.06 per cent of all hospital attendees^2^. The frequency of HIV-2 in the blood donor population at a tertiary referral hospital in southern India between 1998 - 2007 was also similar at 2.8 per cent of all HIV diagnoses (1.3% HIV-2 and 1.5 % HIV-1/2 dual infection)^3^. UNAIDS estimates the number of HIV-2 infected individuals in India to be 2.4 million, but there may well be under-ascertainment^4^. The report from Chiara *et at*^1^ serves as a timely reminder that the problems encountered by the presence of HIV-2 infection, with respect to diagnosis and treatment, are not confined to the more frequently reported cohorts in West Africa and Europe.

In contrast to the devastating pandemic spread of HIV-1, the HIV-2 epidemic has largely been limited to West Africa and countries with colonial links to the subregion, with a striking presence in several ex-Portuguese colonies. Although the lower viral loads and transmissibility of HIV-2 may provide an explanation for this limited geographical distribution, what is more difficult to reconcile is how the overall HIV-2 prevalence in countries such as Guinea-Bissau reached 8-10 per cent in the late 1980s, with up to 20 per cent of those over 40 yr old infected^5^. It is possible that social factors and iatrogenic spread during the Portuguese war of independence may have played a role^6^. An analysis of the emergence of viral population diversity suggested a rapid exponential growth in HIV-2 occurred in this region during this time^7^. It is now clear, however, that in West Africa, the prevalence has stabilised or is reducing^8^, and only time will tell whether this intriguing retroviral infection will disappear altogether.

It is a commonly held view that those infected with HIV-2 progress to AIDS uniformly at a slower rate than their HIV-1 counterparts, yet several studies have demonstrated that this is an oversimplification of the natural history of HIV-2 infection. Over an 18-year period in a well-characterised HIV-2 community cohort in rural Guinea-Bissau, undetectable plasma viral load at baseline predicted both the continued absence of detectable viraemia, as well as survival not appreciably different from HIV-uninfected controls^9^. In contrast, those with high viraemia had approximately 5-fold higher risk of mortality and in clinical settings, HIV-1 and HIV-2-infected individuals matched for viral load, as well as those with CD4 counts below 500/µl, progress to AIDS at similar rates^10^,^11^. Outcomes in HIV-2 infection therefore, appear to be dichotomous, with some individuals remaining asymptomatic elite controllers over approximately two decades, whereas others progress to AIDS and will require the same level of medical care as that afforded to HIV-1 infected patients worldwide.

The study by Chiara *et at*^1^ contains two major aspects worth highlighting. Firstly, it is clear that HIV-2 and HIV-1/2 dual infections are prevalent in patients presenting to clinics in India requiring ART and the lack of discriminatory diagnosis for these infections in national testing algorithms is of great concern. As the clinic acted as a referral centre for groups lacking access to the public system, the exact proportion this represents may be an overestimate (5.3% HIV-2 and 1.34% HIV-1/2 of all HIV diagnoses); but as accurate discrimination from HIV-1 infection is the foundation of appropriate antiretroviral care, this issue cannot be ignored. Due to serological cross-reactivity between HIV-1 and HIV-2, setting up adequate testing algorithms in areas where both viruses circulate can be a challenge, especially in resource poor settings. The authors’ own algorithm appears to be relatively robust, even in the absence of confirmatory PCR, which is often considered the gold standard in the event of dual-seroreactivity. Of some concern though is the high proportion of individuals classified as indeterminate (14.2%) and procedures to resolve these diagnoses either with follow up serological testing or PCR will be vital. We support the authors’ call for increasing availability of discriminatory HIV-1/2 kits at field sites as a pragmatic solution, with access to confirmatory testing where required at referral centres.

Secondly, and of relevance to HIV-2 care globally, are the outcomes of patients on ART. Treatment of HIV-2 is complicated by its well known intrinsic resistance to the ’first-generation’ non-nucleoside reverse transcriptase inhibitors (NNRTIs), nevirapine and efavirenz. In addition, the presence of several natural polymorphisms in the protease gene of HIV-2 corresponds to drug resistance mutations in HIV-1 (*e.g*., M46I), or reduces the genetic barrier to resistance to protease inhibitors (PIs) such as lopinavir (*e.g*., V32I and I47V)^12^. As a result, the options available for first-line HIV-2 ART are limited to either triple nucleoside reverse transcriptase inhibitor (NRTI) or boosted-protease inhibitor (PI)-based regimens using saquinavir (SQV), lopinavir (LPV), darunavir (DRV) or indinavir (IDV). Many of the other PIs used in HIV-1 therapy are less efficacious against HIV-2 *in vitro*^13^. The evidence base available to guide HIV-2 ART choices is restricted and to date there are no reported randomized controlled trials in this field, leaving us to rely largely on small cohort studies and case series for evidence of clinical efficacy. Given the low and reducing prevalence of HIV-2 and its concentration in resource-poor countries, this is perhaps not surprising, but it is an issue that needs to be addressed urgently.

Although the number of ART-naïve HIV-2-infected individuals commencing a triple NRTI regimen (zidovudine, lamuvidine and either tenofovir or abacavir) in the current report^1^ is small, there is a clear warning that such PI-sparing first-line ART for HIV-2 may be suboptimal. This group of patients showed a *decline* in CD4 count over the first 12 months of therapy and were all switched to a boosted-PI regimen. Previous reports have also suggested that triple-NRTI regimens perform poorly in HIV-2-infected patients^14^,^15^ and despite potential benefits such as lower pill burden and reservation of PIs for 2^nd^ line therapy, mounting evidence suggests that this strategy would best be avoided. The remaining HIV-2 infected individuals in this study were treated successfully with indinavir/ritonavir (IDV/r)-based ART and encouragingly showed CD4+ T-cell reconstitution not significantly worse at 6 and 12 months than their HIV-1-infected counterparts, notably in spite of previously receiving ART for erroneously diagnosed HIV-1 at other treatment centres. This is in contrast to some reports demonstrating poorer CD4 recovery in ART treated HIV-2 patients^16^,^17^ and the reason for this discrepancy is not clear. Given the rapid development of resistance mutations in the HIV-2 reverse transcriptase with ineffective therapy^18^,^19^, treatment with an HIV-1 regimen would likely result in accumulation of NRTI resistance. As the current study reports, only 12-month outcomes for HIV-2 infected patients and does not include viral load testing, further investigation is required to conclude that the reported approach is truly safe.

HIV-2 infection is clearly not a global public health problem on the same scale as its more virulent and transmissible cousin, HIV-1, yet this is little consolation to those who are infected with HIV-2 and who are progressing to AIDS. They should be afforded the same standard of clinical care as HIV-1-infected individuals, yet being a minority has resulted in a significant handicap. Chiara and colleagues^1^ highlight the fact that in India, even the first step of incorporating HIV-2 diagnosis into national testing algorithms has not yet been taken. While this problem needs to be tackled nationally in India, given the small size of most individual cohorts, the more daunting task of establishing what constitutes optimum ART in HIV-2 will likely require controlled trials across several cohorts, countries and continents.
